# Advancing Molecular Regulation in Brain Injury Research: Mechanisms, Diagnosis, and Rehabilitation

**DOI:** 10.3390/biom16081194

**Published:** 2026-08-17

**Authors:** Guanglin Zhang, Pavan Thapak

**Affiliations:** Department of Integrative Biology and Physiology, University of California, Los Angeles, CA 90097, USA

Traumatic brain injury (TBI) and non-acquired brain damage represent a catastrophic global health burden, frequently resulting in persistent neurological impairment, long-term cognitive decline, and increased susceptibility to secondary psychiatric disorders. Globally, an estimated 69 million people suffer from TBI, making neurotrauma one of the primary drivers of global morbidity and mortality [1]. The primary mechanical insult of brain injury initiates a prolonged, highly complex secondary injury cascade characterized by blood–brain barrier (BBB) disruption, acute neuroinflammation, excessive glutamate toxicity, ionic imbalance, oxidative stress, and energy deprivation [2]. Despite advances in research and clinical management of acute neurotrauma, effective therapeutic options remain limited. This clinical deficit stems largely from a historical focus on mitigating primary damage rather than targeting the molecular mechanisms that govern secondary degeneration. In this context, exploring the molecular regulation directly or indirectly associated with the pathophysiology and diagnosis of brain injury is essential for uncovering reliable biomarkers, the development of new therapeutic potential, and supporting rehabilitation.

This Special Issue, entitled “Advancing Molecular Regulation in Brain Injury Research: Mechanisms, Diagnosis, and Rehabilitation”, brings together original research and translational synthesis addressing key molecular pathways across various brain injury paradigms including traumatic brain injury (FPI, CCI) and non-acquired brain injury (alcohol-induced injury), as well as comprehensive review articles that provide future directions on bloodstream biomarkers for diagnostic tools in brain injury management.

A core hallmark of secondary brain injury is severe metabolic deprivation and mitochondrial dysfunction, which rapidly deplete cellular energy stores and accelerate apoptotic cascades. The central focus of this issue is the identification of therapeutic targets and molecular interventions that can facilitate recovery and improve outcomes following brain injury. In this regard, Thapak et al. (Contribution 1) demonstrate that TBI triggers profound metabolic deprivation, which is central to secondary pathogenesis. Humanin (HN), a mitochondrial peptide administered within the first 6 h of brain injury, conferred sustained neuroprotective effects that last for three weeks. HN treatment restored bioenergetic homeostasis, attenuated inflammation, suppressed oxidative stress, and enhanced neuronal survival, establishing a crucial translational therapeutic window for clinical testing.

Another major contributor to secondary injury following TBI is glutamate-mediated excitotoxicity. In this issue, Bin Bu et al. (Contribution 2) identify glutamate transporter 1 (GLT-1)—the principal astrocytic transporter responsible for synaptic glutamate clearance—as a promising therapeutic target following TBI. Brain injury caused significant reductions in GLT-1 levels in the hippocampus and frontal cortex. Pharmacological modulation of endocannabinoid signaling using CB1 receptor antagonist AM281 and the monoacylglycerol lipase (MAGL) inhibitor JZL184 restored GLT-1 levels, suppressed neuronal apoptosis, and improved cognitive recovery. Thus, pharmacological modulation of GLT-1 may represent a novel therapeutic strategy for mitigating excitotoxic damage and improving neurological outcomes following TBI.

Expanding beyond TBI, Ming Tong et al. (Contribution 3) investigate the molecular mechanisms underlying alcohol-related brain damage (ARBD). The authors demonstrate that both short- and long-term ethanol exposures disrupt insulin/IGF/Akt-mTOR pathways, which drives progressive cerebral atrophy and white matter myelin loss. These findings highlight the essential role of intact mTOR signaling in maintaining structural neurovascular and oligodendrocyte integrity against chemical neurotoxicity.

This Special Issue highlights the importance of chemokines in the blood–brain barrier (BBB) and microbiota in the gut–brain axis in directing secondary neuroinflammation. A comprehensive review by Qiang Wu et al. (Contribution 4) details the complex and bidirectional functions of major chemokine families (e.g., CCL2/CCR2, CXCL12/CXCR4, CX3CL1/CX3CR1) on the BBB. The authors highlight that elevated concentrations of chemokines (CXCL12 ≥ 2.5 ng/mL; CXCL8 > 10 ng/mL; CCL2 > 3 ng/mL; and CCL5 > 5 ng/mL) trigger the activation of inflammatory pathways, recruitment and infiltration of peripheral immune cells, and microglial polarization, which promotes the intensity of secondary injury. Importantly, these elevated chemokine signatures offer valuable therapeutic targets and diagnostic tools for the monitoring and management of TBI pathogenesis.

Similarly, Tarek Benameur et al. (Contribution 5) explore the growing importance of microbiota-derived extracellular vesicles (EVs) as potential mediators of gut–brain communication following TBI. Their review highlights that EVs encapsulating bioactive molecular cargo, including proteins, lipids, and microRNAs (e.g., miR-124-3p and miR-155), can influence neuroinflammatory responses and promote neuroprotection. These observations provide new insights into EVs as promising candidates for both diagnostic and therapeutic applications in brain injury.

Overall, the studies featured in this Special Issue advance our mechanistic understanding of bioenergetic recovery, excitotoxicity, neurovascular integrity, chemokine-driven BBB dynamics, and gut–brain axis communication underlying brain injury pathology. By bridging basic molecular signaling with translational biomarker discovery, these findings strengthen the foundation for future clinical investigations to validate therapeutic windows and develop reliable diagnostic tools for the management of brain injury.

## References

[1] Dewan M.C., Rattani A., Gupta S., Baticulon R.E., Hung Y.C., Punchak M., Agrawal A., Adeleye A.O., Shrime M.G., Rubiano A.M. (2018). Estimating the global incidence of traumatic brain injury. J. Neurosurg..

[2] Ng S.Y., Lee A.Y.W. (2019). Traumatic Brain Injuries: Pathophysiology and Potential Therapeutic Targets. Front. Cell. Neurosci..

